# Incidence of hepatocellular carcinoma in beta thalassemia: a systematic review and meta-analysis

**DOI:** 10.1016/j.htct.2025.103934

**Published:** 2025-07-31

**Authors:** Marcella Adisuhanto, Alver Prasetya, Alius Cahyadi, Amaylia Oehadian

**Affiliations:** a Department of Internal Medicine, School of Medicine and Health Sciences, Atma Jaya Catholic University of Indonesia, Jakarta, Indonesia; b School of Medicine and Health Sciences, Atma Jaya Catholic University of Indonesia, Jakarta, Indonesia; c Division of Hematology and Medical Oncology, Department of Internal Medicine, Padjadjaran University, Bandung, Indonesia

**Keywords:** Hepatocellular carcinoma, Thalassemia

## Abstract

**Background:**

Current evidence indicates that iron overload increases the risk of hepatocellular carcinoma. However, the incidence of hepatocellular carcinoma in thalassemia is still unclear. This review aims to summarize the current evidence regarding the incidence of hepatocellular carcinoma in thalassemia patients.

**Methods:**

Detailed searches were conducted in several databases, including PubMed, Europe PMC, EBSCOHost, and ProQuest. Keywords such as “thalassemia” and “hepatocellular carcinoma,” along with other relevant synonyms, were used. Articles investigating the incidence of hepatocellular carcinoma in thalassemia patients were included. Pooled estimates were calculated using the DerSimonian Laird inverse-variance random effect model and presented as incidence (%) along with their 95 % confidence intervals and 95 % prediction intervals.

**Results:**

From a total of 318 articles, five studies encompassing a total of 9592 thalassemia patients were included in this study. The cumulative incidence of hepatocellular carcinoma in thalassemia patients was 1.96 % (95 % confidence interval: 0.88 %–4.27 %; prediction interval: 0.12 %–24.74 %; I^2^ = 86.8 %). Of the 139 hepatocellular carcinoma patients, 121 were reported positive for anti-HCV, 78 for HCV RNA, three for HbsAg, and 50 positive for anti-HBV or had past infections. The liver iron concentration and ferritin level ranges in all studies were 2.95–10.5 mg/g and 3.1–2950 µg/L, respectively.

**Conclusions:**

The present meta-analysis demonstrates that the incidence of hepatocellular carcinoma in thalassemia patients was high (1.96 %). It might be caused by liver infection, iron overload, or something else.

## Introduction

Hepatocellular carcinoma (HCC) is the most common form of liver cancer with more than one million people affected each year.^1^ It is also the third cause of cancer-related death worldwide.^2^ There are various risk factors related to HCC such as viral hepatitis, alcoholic liver diseases, and metabolic diseases.^3^ Nowadays iron overload has been linked to the development of HCC. Iron overload induces hepatocyte proliferation, ferroptosis, impaired p53 expression, and mitochondrial iron accumulation that could promote HCC.^4^ There are several causes of iron overload including transfusion dependent thalassemia.^5^

Thalassemia is a condition where there is inadequate production of globin protein leading to ineffective oxygen transport^6^ with 35 % of thalassemia patients being dependent on routine transfusions: this can lead to the development of iron overload if not monitored regularly.^7^ Excessive deposits of iron in various organs can lead to chronic liver disease and thalassemia patients are more exposed to blood-transmitted diseases such as chronic viral hepatitis.^8^

Thalassemia patients are potentially at higher risk for developing HCC compared to the normal population. However, the incidence of HCC in thalassemia is still unclear. This review aims to summarize the current evidence regarding the incidence of HCC in thalassemia patients.

## Methods

Three independent investigators performed detailed searches for relevant studies in several databases including PubMed, Cochrane Controlled Register of Trials (CENTRAL), Europe PMC (medRxiv and bioRxiv), EBSCOHost (Medline), and ProQuest (Gray Literatures) from inception to 30 July, 2023 using keywords such as “thalassemia” and “hepatocellular carcinoma,” along with other relevant synonyms. Articles investigating the incidence of HCC in thalassemia patients were included in this study. There were no restrictions on time or settings. Studies were excluded if they met any of the following criteria: 1) case reports, letters to editors, reviews; 2) non-English articles; or 3) irretrievable full-text articles.

The study selection was done by three authors independently, and disagreement was resolved by the fourth author. Duplicates and irrelevant articles were excluded. The authors screened the titles and abstracts obtained through the search before excluding any work that did not meet the inclusion criteria. Selected studies at this stage were screened further using the full text of the records to determine their eligibility. Any disagreements at each stage of the selection process were resolved by discussion. Data extraction, including author’s name, year of publication, study characteristics, patient characteristics, and outcomes, was input into a web-based word processor.

To assess the risk of bias, all authors independently assessed methodological quality of the studies using the Quality in Prognosis Studies tool.^9^ I-squared statistics were employed to analyze the heterogeneity of the studies. Pooled estimates were calculated using the DerSimonian Laird inverse-variance random effect model and presented as incidence (%) along with the 95 % confidence intervals and 95 % prediction intervals. Sensitivity analysis was done by leave-one-out analysis.

## Results

**Figure 1:**
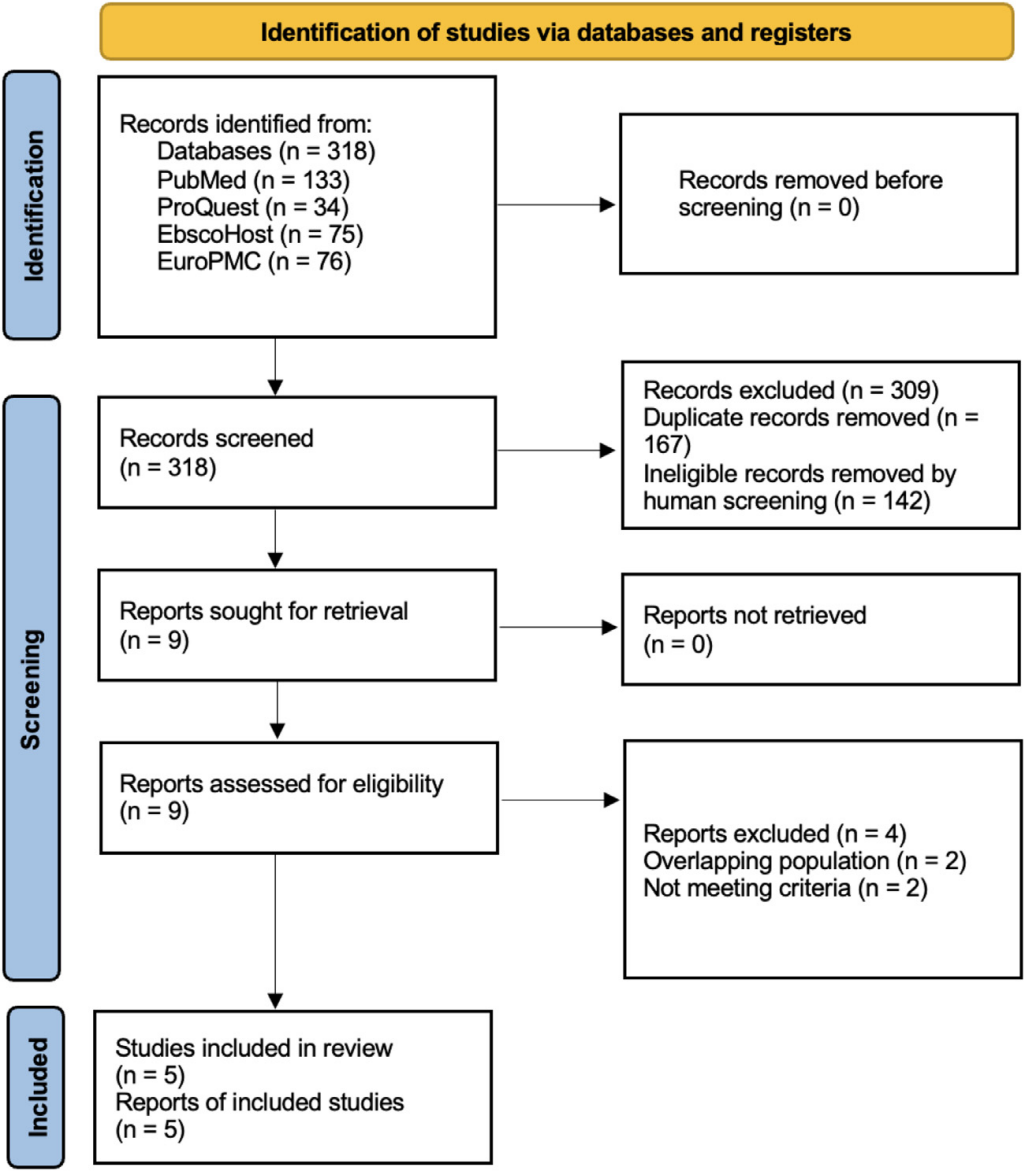
Literature search process and results.

From a total of 318 articles, 167 duplicates and 142 ineligible records were removed. Nine studies were assessed for eligibility resulting in four studies excluded because of overlapping populations and not meeting the study criteria. Five studies, encompassing a total of 9592 thalassemia patients, were included in this study (Figure 1).^10^, ^11^, ^12^, ^13^, ^14^

Of all the patients, 73.8 % (*n* = 7083) had thalassemia major, and 26.1 % (*n* = 2509) had thalassemia intermedia. Three studies from Italy, one study from Iran, and one study from Greece reported HCC incidence rates of from 1.02 % and 2.09 %, 0.6 %, and 7.57 %, respectively. Of the 139 HCC patients, 121 were reported positive for anti-HCV, 78 for HCV RNA, three for HbsAg, and 50 were positive for anti-HBV or had infections. The liver iron concentration (LIC) and ferritin level ranges in all studies were 2.95–10.5 mg/g and 3.1–2950 µg/L, respectively.

**Figure 2:**
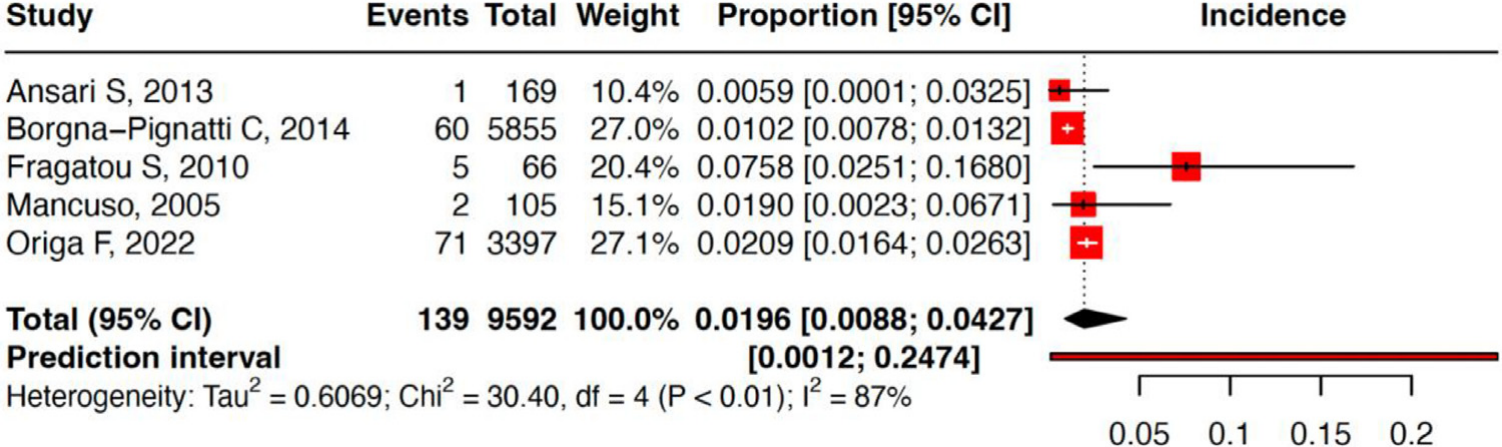
Pooled overall incidence of hepatocellular carcinoma in thalassemia patients.

**Figure 3:**
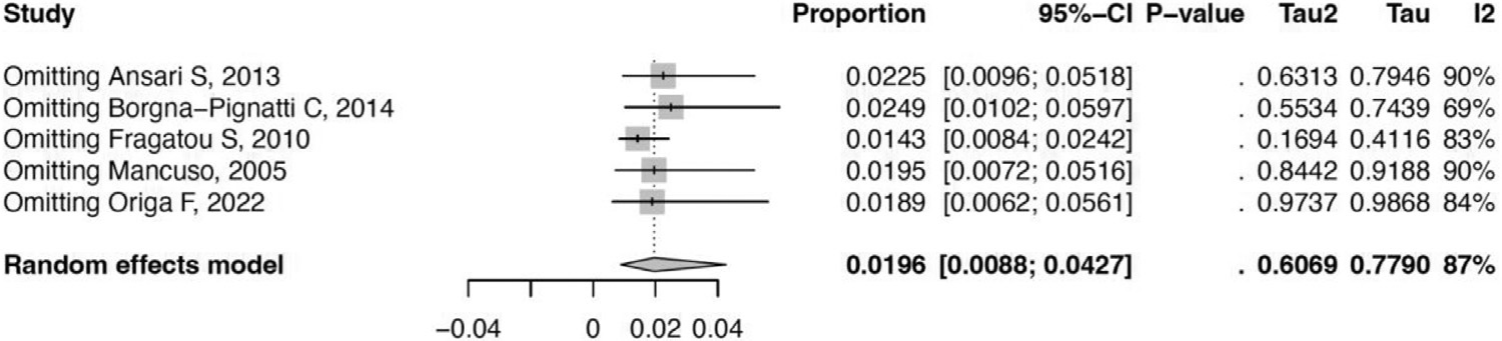
Leave-one-out sensitivity analysis.

**Figure 4:**
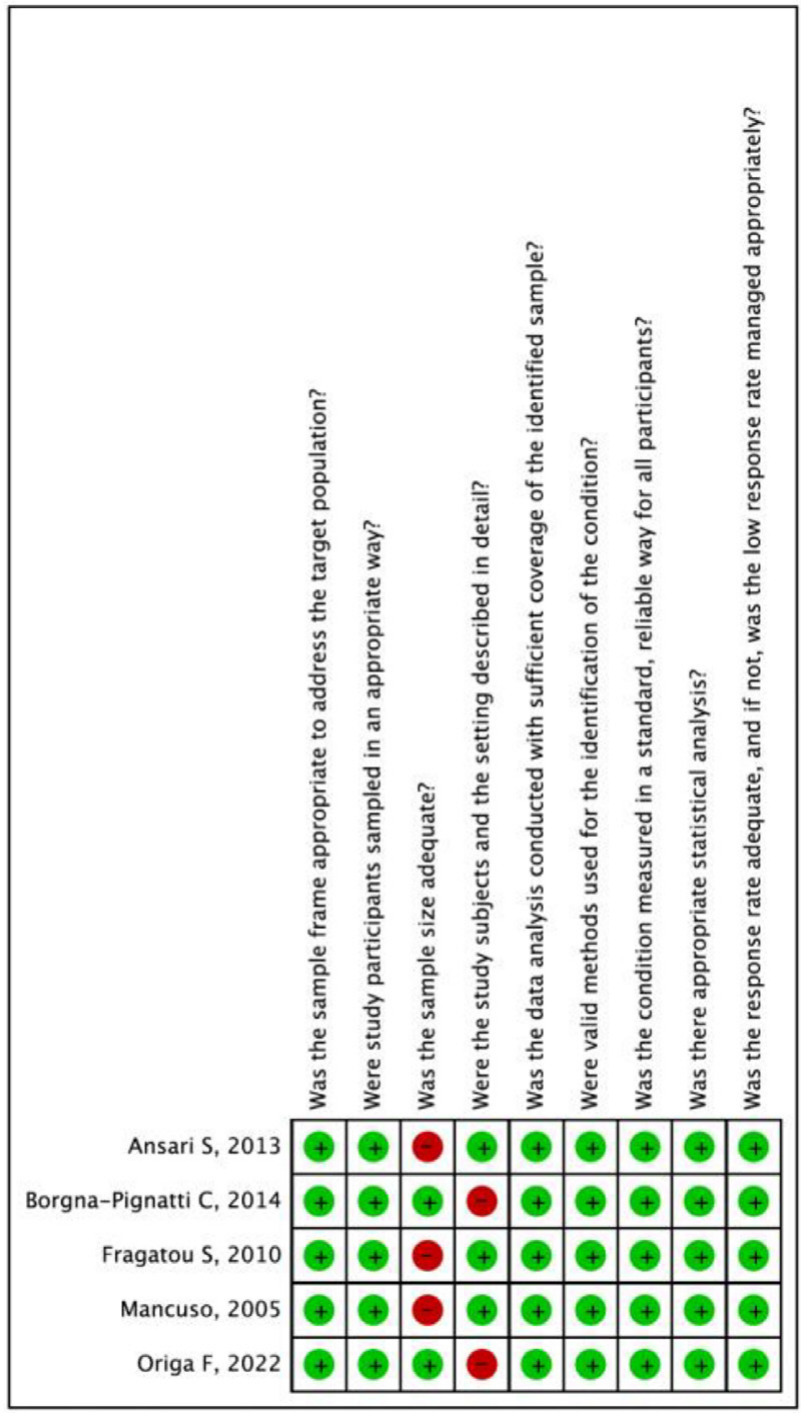
Risk of bias summary for included studies assessed using Joanna Briggs Institute Critical Appraisal Tools.

The cumulative incidence of HCC in thalassemia patients was 1.96 % (95 % confidence interval: 0.88 %–4.27 %) with a prediction interval of 0.12 %–24.74 % and I^2^ of 86.8 %. Sensitivity analysis revealed similar estimates when each study was sequentially removed. This indicates that the results are robust and without inter-studies heterogeneity. Risk of bias assessment using Joanna Briggs Institute Critical Appraisal Tools found that all studies had a low risk of bias (Figure 2, Figure 3, Figure 4, Table 1).

**Table 1:** Characteristics of included studies.

Ref	Author	Year	Setting	No of patients	Other hemoglobinopathies	Total HCC incidence (Thal Only)	Total HCC incidence (All hemoglobinopathies)	HBV/HCV status in Thal patients with HCC	Iron status in Thal patients with HCC	Thal with HBV	Thal with HCV
^10^	Ansari S	2013	Iran	170 (TM = 164; TI = 5; SCD = 1)	SCD = 1	1/169 (0.6 %)	1/170 (0.6 %)	Anti-HCV and HCV RNA (+) = 1/1	NR	–	TM = 164; TI = 5
^13^	Borgna-Pignatti C	2014	Italy	5857 (TM = 4248; TI = 1607 SCD = 2)	SCD = 2	60/5855 (1.02 %)	NR	HBsAg (+) = 3/60; Anti-HBc (+) = 36/60; HBV vaccination = 22/60; Anti-HCV (+) = 52/60; HCV RNA (+) = 43/60; HBV/HCV (-) = 4/60; Unknown serology status = 1/60	TM LIC (median) = 2.95 mg/g; TI LIC (median) = 9 mg/g; TM ferritin (median and peak) = 937 µg/l and 2001 µg/l; TI ferritin (median and peak) = 1181 µg/l and 2950 µg/l	NR	NR
^11^	Fragatou S	2010	Greece	66 (TM = 57; TI = 9)	–	5/66 (7.57 %)	–	Anti-HCV (+) = 2/5; HCV RNA (+) = 2/5 Anti-HBc = 1/5; HBV/HCV (-) = 3/5	TM LIC = 4.9 and 0.215 mg/g; TI LIC = 4.8, 5.2, and 6.9 mg/g; TM ferritin = 18.9 and 3.1 µg/l (1890 and 310 ng/dL); TI ferritin = 6, 13.5, and 14.5 µg/l (600, 1350, and 1450 ng/dL)	–	TM = 23
^14^	Mancuso A	2005	Italy	105 (TM = 35; TI = 70)	–	2/105 (1.90 %)	–	Anti-HCV (+) = 2/2; HCV RNA (+) = 2/2	Iron overload (+) = 2/2; LIC = NR	TI = 2	TM = 28; TI = 18
^12^	Origa F	2022	Italy	4631 (TM = 2579; TI = 818; Others = 1234)	SCD = 815; HbH = 384; Others = 35	71/3397 (2.09 %)	78/4631 (1.68 %)	Anti-HBV = 14/67; HBV DNA = 1/25; Anti-HCV (+) = 64/78; HCV RNA (+) = 30/68 (Serology status including other hemoglobinopathies)	LIC at diagnosis = 5.2 mg/g; LIC peak before diagnosis = 10.5 mg/g; Ferritin at diagnosis (median) = 786 ng/ml (786 µg/l); Ferritin peak before diagnosis = 2704 ng/ml (2704 µg/l)	NR	NR

Thal: thalassemia; TM: thalassemia major; TI: thalassemia intermedia; SCD: sickle cell disease; HBV: hepatitis B virus; HCV: hepatitis C virus; HCC: hepatocellular carcinoma; LIC: liver iron concentration; NR: not reported.

## Discussions

HCC can increase morbidity and mortality in thalassemia population especially when they are 41–50 years old for thalassemia major, and 61–65 years old for thalassemia intermedia. HCC was one of the most frequent solid malignancies in thalassemia patients.^15^ HCC is because of iron overload and/or transfusion-transmitted viral infections, hepatitis B or hepatitis C, immunology abnormality, hydrea use, bone marrow stimulation due to chronic anemia. According to the subject characteristics in this study, most were hepatitis B or hepatitis C positive. Only seven subjects did not have hepatitis B or C. One study did not give the details.^12^

The mean age at diagnosis of HCC was younger than for the non-thalassemia population. This might be due to hemosiderosis as an additional factor for HCC.^16^, ^17^ Iron overload can happen primarily due to the suppression of hepcidin synthesis in the liver, it increases recycled iron released from the reticuloendothelial system and also increases intestinal absorption. It also occurs secondary to regular transfusions especially in thalassemia major patients.^18^ Iron induces toxicity damage which results in genotoxicity, immunological aberrancies, and attenuating cancer immune surveillance.^19^

According to this analysis LIC and ferritin level ranges in all studies were 2.95–10.5 mg/g and 3.1–2950 µg/L. Borna-Pignatti et al. found that three out of four patients without hepatitis B or hepatitis C had high levels of ferritin.^13^ Another study by Maakaron et al. also mentioned two cases of HCC in hepatitis negative patients with thalassemia intermedia with both having high levels of ferritin and liver iron.^20^ These conditions had been studied in other populations such as hereditary hemochromatosis (HH) and iron overload. The researchers found a significant relationship, stating that patients with HH had a 23-fold higher risk of developing HCC compared to healthy individuals.^20^ The annual incidence rate of HCC related liver cirrhosis was 3 %–4 %.^21^

In general, it was believed that HCC was more common in patients with transfusion dependent thalassemia than non-transfusion dependent thalassemia with the milder progression of iron overloading and a lower incidence of chronic viral liver infections being possible explanations.^17^ But there was also another theory related to the difference of iron overload impact between thalassemia major (TM) and intermedia (TI). In TI, similar to genetic haemochromatosis, the iron is absorbed directly from the intestinal tract and loads to hepatocytes. A different process happens in TM. The transfused iron initially goes to Kupffer cells. This different pathway makes the liver iron level in TI higher than in TM which might increase the prevalence of HCC in TI than in TM.^13^

This high iron level, if it happens above the ferritin synthesizing capacity of the cells, may generate reactive oxygen species (ROS) and mutations. Imbalance of immune regulation as another result of iron overload decreases the CD4/CD8 ratio and modulates cytokine activity. Both are responsible for self-defense against viruses and malignant cells. These changes may lead to cancer development.^19^ Iron overload also activates stellate cells and profibrogenic effects of lipid peroxidation, thus accelerating fibrosis to cirrhosis and HCC.^17^

The role of iron in the development of HCC can be prevented by using iron chelation. Some guidelines recommend initiation of chelation therapy in non-transfusion dependent patients with ferritin levels >800 ng/L or LIC >5 mg/g dry weight.^22^ An experimental study by Qian Ba et al. proved that a potent iron chelator can suppress tumor growth of HCC. It reduced available iron, triggering cell-cycle arrest, and apoptosis. An experimental study by Qian Ba et al. proved that iron chelators can suppress tumor growth in HCC. It reduced available iron, triggering cell-cycle arrest, and apoptosis.^23^ The most widely iron chelators used in clinical settings are desferrioxamine (DFO), deferasirox (DFX), and deferiprone.^24^ DFX-DFO combination or DFX as monotherapy have been proven to reduce LIC effectively.^25^

## Conclusions

The present meta-analysis demonstrates that the incidence of HCC in thalassemia patients was high (1.96 %). It might be caused by liver infection, iron overload, or something else. More studies are needed to further estimate the incidence of HCC in thalassemia patients and its pathogenesis.

## Conflicts of interest

The authors declare no conflicts of interest.
